# Antibody-Based Assessment of *Coxiella burnetii* Circulation in Algerian Goat Herds

**DOI:** 10.3390/ani13182926

**Published:** 2023-09-15

**Authors:** Jaqueline T. Bento, Abdeldjalil Dahmane, Sérgio Santos-Silva, Nassiba Reghaissia, Daniela Almeida, João R. Mesquita

**Affiliations:** 1 Department of Biology, Campus de Santiago, University of Aveiro (UA), 3810-193 Aveiro, Portugal; jaquelinetbento@ua.pt; 2 Higher National Veterinary School, Rue Issad Abbes, El Alia, Algiers 16000, Algeria; a.dahmane@etud.ensv.dz; 3 School of Medicine and Biomedical Sciences (ICBAS), University of Porto, 4200-319 Porto, Portugal; up202110051@edu.icbas.up.pt (S.S.-S.); dgomesalmeida23@gmail.com (D.A.); 4 Laboratory of Sciences and Living Techniques, Institute of Agronomic and Veterinary Sciences, University of Souk Ahras, Souk Ahras 41000, Algeria; n.reghaissia@univ-soukahras.dz; 5 Epidemiology Research Unit (EPIUnit), Instituto de Saúde Pública da Universidade do Porto, 4050-600 Porto, Portugal; 6 Laboratory for Integrative and Translational Research in Population Health (ITR), 4050-600 Porto, Portugal

**Keywords:** *Coxiella burnetii*, Algeria, goat, serology

## Abstract

**Simple Summary:**

Q fever, a zoonotic disease caused by the pathogen *Coxiella burnetii*, which can infect both animals and humans, mainly affects ruminant animals such as goats, leading to reproductive problems. Humans can contract the disease by coming into contact with infected animals or their products. In Algeria, Q fever is widespread, but little is known about its presence in domestic goats. This study aimed to analyze *C. burnetii* antibodies in goats from four provinces in Northeastern Algeria. Of the 504 serum samples tested, from 77 herds, 44 showed the presence of *C. burnetii* antibodies. This study found that 35.06% of herds and 8.73% of goats had *C. burnetii* antibodies. Herds with a history of abortions had a particularly high infection rate of 88.9%. This research suggests that *C. burnetii* is widespread in goats and could potentially be transmitted to humans.

**Abstract:**

Q fever is a zoonotic disease caused by *Coxiella burnetii* (*C. burnetii*), a pathogen with a high capability for infection. The disease primarily affects ruminants, leading to reproductive disorders, but can also be transmitted to humans through contact with infected animals or their products. In Algeria, Q fever is endemic, but little is known about the presence and circulation of *C. burnetii* in domestic goats. This study aimed to perform a multicentric serological analysis of *C. burnetii* antibodies in domestic goats from four provinces in the North East Region of Algeria. A total of 504 goat serum samples were collected from 77 herds, and serological analysis was performed using an indirect ELISA. The overall seroprevalence at the herd level was 35.06%, and 8.73% at the individual level. Herds with a history of abortions showed a high seropositivity rate of 88.9%. This research indicates the wide distribution of *C. burnetii* in goats in this region, suggesting the potential for zoonotic transmission to humans. Further studies and monitoring programs are essential to gain a comprehensive understanding of *C. burnetii* epidemiology in Algeria and to prevent or mitigate potential outbreaks. Awareness among practitioners and farmers is crucial to address this public health concern effectively.

## 1. Introduction

Q fever is a zoonotic disease found in numerous locations worldwide [1], caused by *Coxiella burnetii*. This bacterium is a small, nonmotile, Gram-negative pathogen that thrives within host cells and exhibits a notable capability for infection [2,3].

*Coxiella burnetii* has the ability to infect multiple animal species, where ruminants represent the primary animal reservoirs [4,5]. In these animals, Q fever is primarily asymptomatic, but can lead to reproductive disorders, such as late-term abortions, stillbirths and delivery of weak or nonviable newborns [6].

*Coxiella burnetii* is endemic in cattle, sheep, goats, buffaloes, and camels throughout the African continent. However, there are notable variations in its prevalence in different regions. A recent serological study involving 2699 animals in Egypt revealed significant differences in the occurrence of *C. burnetii* between different animal species and regions [7]. Notably, camels showed the highest rates of seropositivity, succeeded by cattle, buffaloes, sheep, and goats. In the Eastern Desert, the seropositive rates of animals were the highest, while the Nile Delta and Western Desert had lower rates. Additionally, pasture-based production systems had lower levels of seropositive animals compared to either nomadic or stationary husbandry. Similarly, in Baringo County, Kenya, nomadic pastoralism was linked to a higher prevalence of *C. burnetii* seropositivity in small ruminants [8]. In Algeria, camels have exhibited high levels of *C. burnetii* seroprevalence [9]. Additionally, a study in Chad showed 80% seroprevalence in camels, which is significantly higher than the rates observed in cattle, goats, and sheep [10].

Serology offers information about the exposure of livestock to *C. burnetii*, but has limited insight on the risk of clinical disease. There are a few studies from Africa that specifically examined *C. burnetii* in ruminant abortion. In Niger, it was discovered that 32% of goats with a history of abortion tested positive for *C. burnetii*, while 29% of non-randomly selected goats without a history of abortion also showed seropositivity for *C. burnetii* [11]. In a study of 109 abortions from Egyptian dairy goats, sheep, and cattle, *C. burnetii* DNA was identified in the placenta and vagina swab sample of one aborted goat [12]. Additionally, in Tunisia, *C. burnetii* DNA was also detected in the birth products and vaginal secretions of small ruminants with a history of abortion [13].

In humans, the main sources of transmission are farm animals, including cattle, goats, and sheep. Animals that are infected with *C. burnetii* release the bacterium through different bodily fluids, including urine, feces, placentas, and milk. However, it is most commonly found in birth products, often leading to miscarriages, thereby contributing to the spread of the disease [4,14,15]. In a study conducted in Gambia consisting of small ruminants and humans, direct evidence showed that the presence of *C. burnetii*-positive animals was a significant risk factor in human infection [16]. Moreover, in Northern Kenya, risk factors for *C. burnetii* seropositivity among febrile patients were identified as exposure to cattle, goats, animals slaughter, or the consumption of raw milk products [17]. Another study in Egypt showed that residents in close contact with animals reported a high overall seroprevalence, with grater seropositivity observed among rural residents compared to urban residents [18]. Likewise, in Algeria, being a rural inhabitant was identified as a risk factor for *C. burnetii* seropositivity [19].

Human infection usually occurs from inhaling aerosols of animal origin contaminated with *C. burnetii*, but oral transmission by ingestion of contaminated dairy products is also possible, as well as through sexual and vertical transmission [20,21,22]. Q fever in humans can result in an acute illness that leads to pneumonia, hepatitis, and a self-limited condition [20].

In Algeria, Q fever is present as an endemic disease, following the initial cases reported in Algiers in 1948 and subsequent outbreaks in Batna within the French army in both 1955 and 1957, as well as in Tlemcen, also involving the army, in 1958 [23]. Moreover, a more recent study shows a seroprevalence of 15.5% in people from an agropastoral region with 1,056,489 inhabitants and an estimated livestock number of about 574,000 head in the Wilaya of Setif in Algeria [19]. However, little is known about the presence and circulation of *C. burnetii* main animal reservoirs, particularly those domestic and consequently, in close contact to potentially susceptible humans.

As such, the objective of this study was to perform a multicentric serological analysis of *C. burnetii* antibodies in domestic goats, aiming to obtain a comprehensive understanding of the spread of *C. burnetii* in Northeastern Algeria.

## 2. Materials and Methods

### 2.1. Sampling

**Figure 1:**
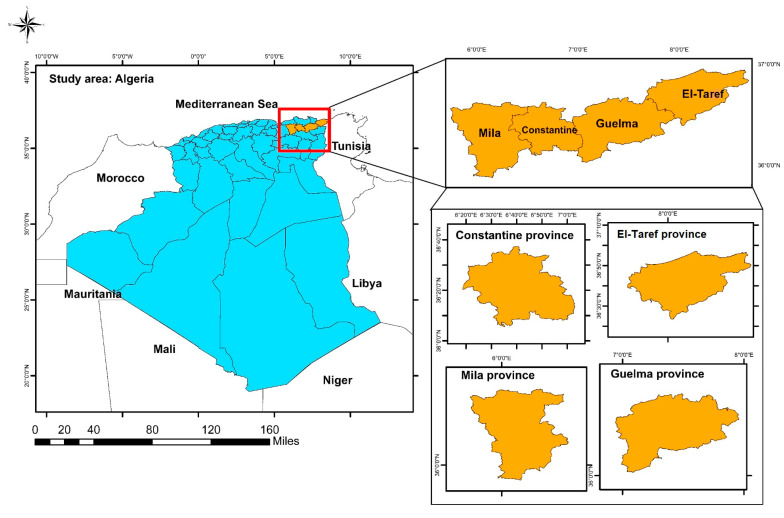
Map of the study area indicating the municipalities of goat herds submitted to the epidemiology investigation for Q fever, the color orange represents the origin provinces of the samples and the blue color the remaining areas of Algeria.

The study was carried out in four provinces of Northeastern Algeria: Mila, Constantine, Guelma, and El-Taref (Figure 1).

Mila lies inland, about 82 km from the Mediterranean coast. The district is characterized by a varied relief and presents two large distinct zones: to the north, mountains and hills (M’sid, Aicha, Zouagha, and El-Halfa); and to the south, the plains and highlands with an area of 3481 km^2^. The region has a Mediterranean climate with hot, dry summers and cold, wet winters. The climate is humid in the north, sub-humid to semi-arid in the center, and semi-arid in the south. The rainfall varies between 600 and 900 mm in the north of the province (920 mm on the mount of Msid Aicha), between 400 and 600 in the center, and less than 400 mm in the south. In the summer, the temperature varies between 25 °C and 40 °C, and the average winter temperatures range from −2 °C to 12 °C.

Constantine is situated on a plateau 698 m above sea level; the area has a Mediterranean climate with hot, dry summers, and cold moist winters. In summer, temperature ranges between 25 °C and 40 °C. The average temperatures in winter lie between 0 °C and 13 °C. The annual rainfall varied between 500 and 700 mm in the course of this study.

The territory of Guelma province is characterized by a sub-humid climate in the center and the north, and semi-arid towards the south. This climate is mild and rainy in winter and warm in summer. The temperature varies from 4 °C in winter to more than 35 °C in summer. The geography of the district is characterized by a diversified relief (mountains, plains and plateaus, hills, and foothills) which mainly retains significant forest cover and the passage of Seybouse River which constitutes the main watercourse.

The El-Taref province is located in the far North East Region of Algeria along the Tunisian border. The climate is generally humid. The humidity decreases from north to south in the following way: a coastal zone that presents a hot and humid climate; a mountainous area that occupies most of the region and has a mild humid climate in the north and cool in the south. The annual precipitation rate is 900 mL to 1200 mL.

An appropriate number of goats were sampled by a simple random sampling method. In selecting the municipalities and properties that participated in this study, the division of the state, ease of access, convenience, and availability of producers were considered. The goats were randomly selected from males and females, apparently healthy, of different zootechnical patterns, and were over three months old. Also, the number of goats taken from each farm was defined based on the total number of animals to have a representative sample of at least 10% of all individuals in each farm visited.

During visits to the properties, a structured questionnaire was administered to each farmer under the supervision of the principal investigator to obtain the epidemiological data. The questionnaires consisted of several closed questions about the sex, age, herd size, and abortion (Supplementary Table S1).

Serum was obtained from 504 goats sampled from January 2020 to October 2022. Samples were collected from 77 herds, from rural and suburban environments. From these, 330 were females (137 of them with a history of abortion), and 174 were males. A total of 201 samples came from animals under 2 years of age, 225 from animals between 2 and 5 years, and 78 from animals over 5 years of age.

Briefly, blood samples were collected by venipuncture of the jugular vein into sterile-labeled vacutainer tubes without additives (BD Vacutainer Systems, Plymouth, UK). Blood tubes were kept at 4 °C and transferred immediately to the laboratory. The collected blood samples were centrifuged at 1800× *g* for 10 min, and the sera were separated and frozen at −20 °C until analysis.

### 2.2. Serological Analysis

All sera (*n* = 504) were examined for the presence of anti-*C. burnetii* IgG antibodies using a commercial indirect ELISA, ID Screen Q Fever Indirect Multi-species Kit (IDvet™, Montpellier, France), following the instructions provided by the manufacturer. ELISA’s sensitivity and specificity have been shown to be 100% (IDvet™, internal validation report). Results were expressed as a percentage of the optical density (OD) reading of the test, calculated as %OD = 100 × (OD sample − OD Negative Control)/(OD Positive Control − OD Negative Control), where OD represents the measured OD. An animal was considered positive (ELISA+) when its %OD had a value between 50% and 80%; highly positive (ELISA++) for values greater than 80%; doubtful for values between 40% and 50%; and negative (ELISA−) for values lower than 40%.

### 2.3. Statistical Analysis

Data obtained from the analysis of sera with ELISA were used to calculate seroprevalence values specific to the population and geographic locations (provinces). To assess differences between groups, the Chi-square test was employed (GraphPad Prism version 5.04; GraphPad Software Inc., La Jolla, CA, USA). A statistically significant result was considered if the *p* value was <0.05. The association between the detection of anti-*C. burnetii* IgG and the variables comprising gender, herd, abortion, herd size, province, and climate were evaluated by binomial logistic regression (univariate) and multinomial logistic regression (multivariate) analysis using IBM SPSS Statistics 28.0.0.0. Confidence interval was established at 95%.

## 3. Results

Overall, 27 out of the 77 herds tested positive for IgG anti-*C. burnetii*, either with or without cases of abortion, resulting in a herd-level seroprevalence rate of 35.06%. Separately, a total of 44 goat samples were found to have *C. burnetii* antibodies, which represent a seroprevalence of 8.73% (95% CI: 6.41–11.54) for anti-*C. burnetii* IgG in goats across the four provinces of the North East Region of Algeria (Mila, Constantine, Guelma, and El-Taref) (Table 1). Among these 44 positive goats, 24 (54.55%, 95% CI: 38.85–69.61) were classified as low-positive and 20 (45.45%, 95% CI: 30.39–61.15) were considered strong-positive. Furthermore, a high seropositivity in herds with a previous history of abortions was observed (88.9%), indicating that 88.9% of the positive herds had documented cases of abortions among their animals. Additionally, 14 out of 34 (41.2%) positive females had a history of abortion. Analyzing the distributions according to provinces, *C. burnetii* antibodies were detected in 14 out of 218 goats in the Mila province (6.42%, 95% CI: 3.55–10.54), of which 10 (71.43%, 95% CI: 41.90–91.61) were low-positives and 4 (28.57%, 95% CI: 8.39–58.10) were strong-positives. In the Constantine province, out of 142 goats, 23 (16.20%, 95% CI: 10.55–23.31) had *C. burnetii* antibodies, with 13 (56.52%, 95% CI: 34.49–76.81) classified as low-positives and 10 (43.48%, 95% CI: 23.19–65.51) as strong-positive. In the Guelma province, out of 118 goats, 6 (5.08%, 95% CI: 1.89–10.74) had *C. burnetii* antibodies, of which 1 (16.67%, 95% CI: 0.42–64.12) was low-positive and 5 (83.33%, 95% CI: 35.88–99.58) were strong-positive. Only 1 out of 26 goats from the El-Taref province (3.85%, 95% CI: 0.10–19.64) was classified as strong-positive (100%, 95% CI: 2.5–100). Moreover, a seroprevalence of 16.05% was observed in the suburban environment, whereas the rural environment showed a seroprevalence of 7.33%.

Regarding univariate and multivariate analysis, no significance was found for the variables comprising sex, herd, abortion, herd size, climate, and province, showing no significant association to the detection of anti-*C. burnetii* IgG (Table 2).

## 4. Discussion

Q fever, a zoonotic illness caused by *C. burnetii* present in various locations worldwide, can affect several animal species, including humans [2,4]. The epidemiology and evolutionary aspects of animal Q fever have received limited attention in most countries, including Algeria. Often, professionals do not suspect the disease even after observing abortion cases, leading to Q fever tests not being a routine part of abortion case differential diagnosis [24].

In this study, a serological analysis of *C. burnetii* antibodies in goat samples was performed, aiming to obtain a comprehensive understanding of the spread of *C. burnetii* across goats, a major animal reservoir, in the Northern Region of the country. Overall seroprevalence rate at the herd level was 35.06%, 27/77 herds tested positive for IgG anti-*C. burnetii*, with or without abortion events. This rate is higher than that reported in a similar study conducted in domestic goat herds from the USA (8.6%) [25]. The seroprevalence rate at the individual level in this study was 44 (8.73%), out of the total 504 tested animals. Other studies on small ruminants (sheep and goats) from Algeria [24] and Egypt [26] showed higher seroprevalence rates (14% and 18.4%, respectively) when compared to our results. These discrepancies should be analyzed with care since our study has a higher sampling size when compared to the others, and also a different indirect ELISA test was used. Moreover, the results may also be explained due to the choice of farms and subjects tested, based on the history of abortions between the different studies.

The level of *C. burnetii* seroprevalence among ruminant herds is regarded as a valuable indicator for investigating its presence in the human population [3]. In this sense, our study suggests that Q fever is widely spread throughout the North East Region of Algeria. Furthermore, in our study, a high seropositivity was observed in herds with a history of abortions (88.9%), meaning that 88.9% of the positive herds had animals with registered abortions. This aligns with findings from another study involving small ruminant flocks in Algeria, which reported an 80% seropositivity rate [24]. These results were observed in previous studies from Spain and France [27,28], where Q fever was linked to abortions, although no statistical significance was observed when considering the abortion variable at the individual level. Separately, 14 out of the 34 (41.2%) positive females had an abortion, which may suggest a link between *C. burnetii* infection and abortion. Although, the difference between positive females and males was not statistically significant, there are several studies showing that being female was found to be a significant risk factor, having more probability of contracting anti-*C. burnetii* IgG compared to males [29,30]. Nevertheless, further studies should be conducted in order to understand if this pattern persists or changes in Algeria.

When comparing anti-*C. burnetii* presence in the collected goat samples according to the province distribution, a higher seroprevalence was observed in the Constantine province (16.20%) when comparing to the other provinces of the North East Region of Algeria, more specifically, in Mila (6.42%), Guelma (5.08%), and El-Taref (3.85%). Mila, Constantine, Guelma, and El-Taref experience different types of climates, specifically, Mila and Constantine experience a Mediterranean climate, Guelma a sub-humid climate, and El-Taref a humid one. In Algeria, sheep are the dominant species among ruminants, constituting 80% of the total estimated livestock population, with more than 25 million individuals, including 12 million ewes. Following sheep, goats are the second-most common species, accounting for 13% of the population, and 58% of them are females. During the summer seasons, transhumance and nomadism towards the North East and North West Regions become necessary, particularly from May to September when the pastures can no longer sustain the flocks [24]. This is also true towards the North East and the South East Regions, where a high individual level of anti-*C. burnetti* antibodies was observed previously [9]. The practice of semi-extensive husbandry in these provinces, which permits sheep to use common pastures during the day, may contribute to a higher prevalence of anti-*C. burnetii* antibodies in these areas. This is because spore-like forms of the bacterium, which can survive for extended periods in the soil, could potentially infect the goats, explaining the circulation of *C. burnetii* in this particular region. Interestingly, a higher seroprevalence was observed in the suburban environment (16.05%) when compared to rural environments (7.33%). The close proximity of humans to goat herds in suburban settings might play a role in the transmission of the disease from animals to humans, posing a concern for public health. Further studies describing the circulation of *C. burnetii* in these types of settings should be conducted to better understand the extent of the risk and to develop targeted strategies for disease prevention and control, safeguarding both human and animal health.

Importantly, no significant differences were found between herds, herd size, climate, and province. These findings suggest that these factors may not have a substantial impact on the variables being investigated, which leads to the necessity for further exploration of other potential factors that might influence the observed outcomes.

To date, only two small ruminant seroprevalence studies have been performed in Algeria, collecting sera from sheep and goats [24,31]. Interestingly, one study from the North Central Region of Algeria [31] showed a global individual seroprevalence of 24.9%, which is higher than the seroprevalence detected in our study. This discrepancy may indicate the spread of *C. burnetii*, especially since both studies used the same enzyme immunoassays. However, these data should be analyzed with care because the aforementioned study was conducted in a different region of the country and only involved sheep, which may also contribute to the observed differences.

The primary objective of this study was to evaluate the presence of anti-*C. burnetii* IgG antibodies in goats from across four provinces of the North East Region of Algeria. The ELISA results unequivocally indicate that the tested animals have been exposed to the infectious agent. Moreover, this study demonstrates the circulation of *C. burnetii* not only in females that experienced abortions, but also in those that had normal deliveries. Furthermore, there is a possibility that these animals might be chronically infected, leading to the shedding of bacteria during future pregnancies, contributing to environmental contamination and the subsequent spread of the infection. The limited understanding of Q fever raises the risk of infection for both livestock and humans. Hence, it is crucial to raise awareness among practitioners, farmers, and testing laboratories to address this issue effectively.

## 5. Conclusions

In conclusion, although seroprevalence in this study seems low when compared to others, the agent appears to be distributed across the North East Region of Algeria and hence alerts for the possibility of zoonotic transmission. The close proximity of humans to goat herds could be a factor in the zoonotic transfer of this disease. Although the initial findings of this study indicate a relatively low Q fever seroprevalence in the area, there is a crucial requirement to gain a more comprehensive understanding of *C. burnetii* epidemiology in Algeria. Establishing monitoring programs for sentinel herds could aid in the prevention or reduction of the impact of potential epidemics.

## Figures and Tables

**Table 1:** Screening for anti-*Coxiella burnetii* IgG antibodies in 504 goats from all four provinces of Algeria.

Province	Anti-*C. burnetii* Pos/Total: No. (%; CI)	Anti-*C. burnetii* Low Pos/Region Total Pos: No. (%; CI)	Anti-*C. burnetii* Strong Pos/Region Total Pos: No. (%; CI)
Mila	14/218 (6.42%; 3.55–10.54)	10/14 (71.43%; 41.90–91.61)	4/14 (28.57%; 8.39–58.10)
Constantine	23/142 (16.20%; 10.55–23.31)	13/23 (56.52%; 34.49–76.81)	10/23 (43.48%; 23.19–65.51)
Guelma	6/118 (5.08%; 1.89–10.74)	1/6 (16.67%; 0.42–64.12)	5/6 (83.33%; 35.88–99.58)
El-Taref	1/26 (3.85%; 0.10–19.64)	0/1 (0; 0–97.5)	1/1 (100%; 2.5–100)
Total	44/504 (8.73; 6.41–11.54)	24/44 (54.55%; 38.85–69.61)	20/44 (45.45%; 30.39–61.15)

**Table 2:** Univariate and multivariate analysis for the variables comprising herd size, climate, province, gender, and abortion.

Variable	Univariate Analysis cOR (95% CI)/*p* Value	Multivariate Analysis aOR (95% CI)/*p* Value
Herd Size	0.561	0.507
1–10	1.46 (0.18–11.67)/0.72	1.19 (0.137–10.32)/0.876
11–30	1.36 (0.71–2.6)/0.355	1.74 (0.20–14.93)/0.614
>31	Ref.	Ref.
Climate	0.059	0.067
Humid	Ref.	Ref.
Sub-humid	2.24 (1.12–4.49)/0.059	2.17 (1.01–4.63)/0.056
Semi-arid	0.325 (0.041–2.56)/0.286	0.54 (0.059–4.93)/0.585
Province	0.055	0.057
Mila	1.72 (0.22–13.61)/0.609	1.3 (0.15–11.28)/0.808
Constantine	4.83 (0.62–37.46)/0.132	2.88 (0.324–25.52)/0.343
Guelma	1.34 (0.15–11.62)/0.791	0.9 (0.086–9.36)/0.926
El-Taref	Ref.	Ref.
Gender		
Male	Ref.	Ref.
Female	1.88 (0.91–3.91)/0.089	2.12 (0.93–4.82)/0.074
Abortion	0.256	0.166
No	0.542 (0.24–1.15)/0.108	0.529 (0.24–1.19)/0.125
Yes	0.914 (0.44–1.91)/0.811	1.19 (0.56–2.54)/0.648
NA	Ref.	Ref.

Ref.—reference value; CI—confidence interval.

## Data Availability

The data presented in this study are available on request from the corresponding author.

## Supplementary Materials

The following supporting information can be downloaded at: https://www.mdpi.com/article/10.3390/ani13182926/s1, Table S1: Sampling information of the 504 goat sera samples collected from Algeria.
