# Supplementary material for: Antibody-Based Assessment of Coxiella burnetii Circulation in Algerian Goat Herds
Source: Animals (Basel). 2023 Sep 15;13(18):2926. doi: 10.3390/ani13182926 (PMC10525101; doi:10.3390/ani13182926)
Supplement: Supplementary file 1 [file animals-13-02926-s001.zip › animals-2566676-supplementary.pdf]

Supplementary Table S1. Sampling information of the 504 goat sera samples collected from Algeria.

N° sample	Sex	Age	Herd Size	Abortion	Province	Environement	Climate
1	M	<B	(+31)	0	Mila	Suburban	Mediterranean
2	F	C	(+31)	A	Mila	Suburban	Mediterranean
3	M	<B	(+31)	0	Mila	Suburban	Mediterranean
4	F	B	(+31)	NA	Mila	Suburban	Mediterranean
5	F	B	(+31)	A	Mila	Suburban	Mediterranean
6	M	B	(+31)	0	Mila	Suburban	Mediterranean
7	F	<B	(+31)	NA	Mila	Suburban	Mediterranean
8	M	<B	(+31)	0	Mila	Suburban	Mediterranean
9	M	C	(+31)	0	Mila	Suburban	Mediterranean
10	F	<B	(+31)	A	Mila	Suburban	Mediterranean
11	M	B	(+31)	0	Mila	Suburban	Mediterranean
12	F	<B	(+31)	NA	Mila	Suburban	Mediterranean
13	F	B	(1-10)	A	Mila	Rural	Mediterranean
14	M	B	(1-10)	0	Mila	Rural	Mediterranean
15	F	C	(1-10)	NA	Mila	Rural	Mediterranean
16	F	B	(1-10)	A	Mila	Rural	Mediterranean
17	F	<B	(1-10)	NA	Mila	Rural	Mediterranean
18	M	<B	(11-30)	0	Mila	Rural	Mediterranean
19	M	<B	(11-30)	0	Mila	Rural	Mediterranean
20	F	B	(11-30)	A	Mila	Rural	Mediterranean
21	F	B	(11-30)	A	Mila	Rural	Mediterranean
22	F	<B	(11-30)	A	Mila	Rural	Mediterranean
23	F	<B	(11-30)	NA	Mila	Rural	Mediterranean
24	F	B	(11-30)	A	Mila	Rural	Mediterranean
25	F	B	(11-30)	A	Mila	Rural	Mediterranean
26	F	<B	(11-30)	A	Mila	Rural	Mediterranean
27	F	<B	(11-30)	NA	Mila	Rural	Mediterranean
28	M	<B	(11-30)	0	Mila	Rural	Mediterranean
29	F	B	(11-30)	A	Mila	Rural	Mediterranean
30	F	B	(11-30)	NA	Mila	Rural	Mediterranean
31	F	<B	(11-30)	A	Mila	Rural	Mediterranean
32	F	<B	(11-30)	NA	Mila	Rural	Mediterranean
33	M	<B	(1-10)	0	Mila	Rural	Mediterranean
34	F	<B	(1-10)	NA	Mila	Rural	Mediterranean
35	F	<B	(1-10)	NA	Mila	Rural	Mediterranean
36	F	<B	(1-10)	A	Mila	Rural	Mediterranean
37	M	<B	(11-30)	0	Mila	Rural	Mediterranean
38	F	B	(11-30)	NA	Mila	Rural	Mediterranean
39	F	<B	(11-30)	A	Mila	Rural	Mediterranean
40	F	<B	(11-30)	A	Mila	Rural	Mediterranean
41	M	B	(11-30)	0	Mila	Rural	Mediterranean
42	F	<B	(11-30)	NA	Mila	Rural	Mediterranean
43	F	B	(11-30)	A	Mila	Rural	Mediterranean
44	F	B	(11-30)	A	Mila	Rural	Mediterranean
45	F	C	(11-30)	NA	Mila	Rural	Mediterranean
46	M	B	(11-30)	0	Mila	Rural	Mediterranean
47	F	<B	(11-30)	A	Mila	Rural	Mediterranean
48	F	<B	(11-30)	A	Mila	Rural	Mediterranean
49	F	B	(11-30)	A	Mila	Rural	Mediterranean

50	M	B	(11-30)	0	Mila	Rural	Mediterranean
51	M	<B	(11-30)	0	Mila	Rural	Mediterranean
52	M	<B	(11-30)	0	Mila	Rural	Mediterranean
53	F	C	(11-30)	NA	Mila	Rural	Mediterranean
54	F	B	(11-30)	A	Mila	Rural	Mediterranean
55	F	<B	(11-30)	A	Mila	Rural	Mediterranean
56	F	C	(11-30)	A	Mila	Rural	Mediterranean
57	M	B	(11-30)	0	Mila	Rural	Mediterranean
58	F	B	(11-30)	A	Mila	Rural	Mediterranean
59	F	B	(11-30)	A	Mila	Rural	Mediterranean
60	M	C	(11-30)	0	Mila	Rural	Mediterranean
61	M	C	(11-30)	0	Mila	Rural	Mediterranean
62	F	B	(11-30)	NA	Mila	Rural	Mediterranean
63	M	<B	(11-30)	0	Mila	Rural	Mediterranean
64	F	<B	(11-30)	NA	Mila	Rural	Mediterranean
65	F	B	(11-30)	A	Mila	Rural	Mediterranean
66	F	B	(11-30)	A	Mila	Rural	Mediterranean
67	M	B	(1-10)	0	Mila	Suburban	Mediterranean
68	F	<B	(1-10)	A	Mila	Suburban	Mediterranean
69	F	<B	(1-10)	A	Mila	Suburban	Mediterranean
70	M	B	(1-10)	0	Mila	Suburban	Mediterranean
71	M	C	(1-10)	0	Mila	Suburban	Mediterranean
72	F	<B	(11-30)	A	Mila	Rural	Mediterranean
73	F	C	(11-30)	NA	Mila	Rural	Mediterranean
74	M	C	(11-30)	0	Mila	Rural	Mediterranean
75	F	B	(11-30)	NA	Mila	Rural	Mediterranean
76	M	<B	(11-30)	0	Mila	Rural	Mediterranean
77	F	<B	(11-30)	A	Mila	Rural	Mediterranean
78	F	B	(11-30)	NA	Mila	Rural	Mediterranean
79	F	B	(1-10)	A	Mila	Rural	Mediterranean
80	F	<B	(1-10)	NA	Mila	Rural	Mediterranean
81	F	B	(1-10)	NA	Mila	Rural	Mediterranean
82	F	<B	(1-10)	NA	Mila	Rural	Mediterranean
83	F	B	(1-10)	A	Mila	Rural	Mediterranean
84	F	C	(1-10)	A	Mila	Rural	Mediterranean
85	M	<B	(1-10)	0	Mila	Rural	Mediterranean
86	M	B	(1-10)	0	Mila	Rural	Mediterranean
87	M	<B	(1-10)	0	Mila	Rural	Mediterranean
88	M	B	(1-10)	0	Mila	Rural	Mediterranean
89	F	B	(1-10)	A	Mila	Rural	Mediterranean
90	F	B	(1-10)	NA	Mila	Rural	Mediterranean
91	M	<B	(1-10)	0	Mila	Rural	Mediterranean
92	M	B	(1-10)	0	Mila	Rural	Mediterranean
93	F	B	(1-10)	NA	Mila	Rural	Mediterranean
94	F	C	(1-10)	A	Mila	Rural	Mediterranean
95	F	C	(1-10)	NA	Mila	Suburban	Mediterranean
96	F	C	(1-10)	NA	Mila	Suburban	Mediterranean
97	M	B	(1-10)	0	Mila	Suburban	Mediterranean
98	M	<B	(11-30)	0	Mila	Rural	Mediterranean
99	F	B	(11-30)	NA	Mila	Rural	Mediterranean

100	F	<B	(11-30)	A	Mila	Rural	Mediterranean
101	F	C	(11-30)	A	Mila	Rural	Mediterranean
102	M	<B	(11-30)	0	Mila	Rural	Mediterranean
103	M	C	(11-30)	0	Mila	Rural	Mediterranean
104	F	C	(11-30)	A	Mila	Rural	Mediterranean
105	F	C	(11-30)	NA	Mila	Rural	Mediterranean
106	F	<B	(1-10)	NA	Mila	Suburban	Mediterranean
107	F	B	(1-10)	A	Mila	Suburban	Mediterranean
108	M	B	(1-10)	0	Mila	Suburban	Mediterranean
109	M	<B	(1-10)	0	Mila	Suburban	Mediterranean
110	F	C	(1-10)	A	Mila	Suburban	Mediterranean
111	F	C	(1-10)	NA	Mila	Rural	Mediterranean
112	F	B	(1-10)	NA	Mila	Rural	Mediterranean
113	M	C	(1-10)	0	Mila	Rural	Mediterranean
114	M	B	(1-10)	0	Mila	Rural	Mediterranean
115	F	<B	(1-10)	NA	Mila	Rural	Mediterranean
116	M	B	(1-10)	0	Mila	Suburban	Mediterranean
117	F	<B	(1-10)	A	Mila	Suburban	Mediterranean
118	M	B	(1-10)	0	Mila	Suburban	Mediterranean
119	F	C	(1-10)	NA	Mila	Suburban	Mediterranean
120	F	B	(1-10)	A	Mila	Suburban	Mediterranean
121	F	B	(1-10)	A	Mila	Rural	Mediterranean
122	F	C	(1-10)	NA	Mila	Rural	Mediterranean
123	M	B	(1-10)	0	Mila	Rural	Mediterranean
124	F	B	(1-10)	A	Mila	Rural	Mediterranean
125	F	<B	(1-10)	A	Mila	Rural	Mediterranean
126	F	<B	(1-10)	NA	Mila	Rural	Mediterranean
127	F	B	(1-10)	A	Mila	Rural	Mediterranean
128	F	C	(1-10)	A	Mila	Rural	Mediterranean
129	M	B	(1-10)	0	Mila	Rural	Mediterranean
130	M	B	(1-10)	0	Mila	Rural	Mediterranean
131	M	<B	(1-10)	0	Mila	Rural	Mediterranean
132	M	C	(1-10)	0	Mila	Rural	Mediterranean
133	F	B	(1-10)	0	Mila	Rural	Mediterranean
134	M	C	(1-10)	0	Mila	Rural	Mediterranean
135	F	B	(1-10)	A	Mila	Rural	Mediterranean
136	F	B	(1-10)	A	Mila	Rural	Mediterranean
137	F	<B	(1-10)	NA	Mila	Rural	Mediterranean
138	M	B	(11-30)	0	Constantine	Rural	Mediterranean
139	M	C	(11-30)	0	Constantine	Rural	Mediterranean
140	F	B	(11-30)	NA	Constantine	Rural	Mediterranean
141	F	<B	(11-30)	NA	Constantine	Rural	Mediterranean
142	F	B	(11-30)	NA	Constantine	Rural	Mediterranean
143	F	B	(11-30)	NA	Constantine	Rural	Mediterranean
144	M	<B	(11-30)	0	Constantine	Rural	Mediterranean
145	M	<B	(1-10)	0	Constantine	Rural	Mediterranean
146	M	<B	(1-10)	0	Constantine	Rural	Mediterranean
147	F	<B	(1-10)	A	Constantine	Rural	Mediterranean
148	F	B	(1-10)	A	Constantine	Rural	Mediterranean
149	M	B	(1-10)	0	Constantine	Rural	Mediterranean

150	F	B	(1-10)	A	Constantine	Rural	Mediterranean
151	F	<B	(1-10)	NA	Constantine	Rural	Mediterranean
152	F	B	(1-10)	NA	Constantine	Rural	Mediterranean
153	F	B	(1-10)	NA	Constantine	Rural	Mediterranean
154	F	<B	(1-10)	A	Constantine	Rural	Mediterranean
155	M	<B	(1-10)	0	Constantine	Rural	Mediterranean
156	M	<B	(1-10)	0	Constantine	Rural	Mediterranean
157	F	<B	(1-10)	A	Mila	Rural	Mediterranean
158	F	B	(1-10)	NA	Mila	Rural	Mediterranean
159	F	<B	(1-10)	NA	Mila	Rural	Mediterranean
160	M	B	(1-10)	0	Mila	Rural	Mediterranean
161	F	C	(1-10)	NA	Constantine	Suburban	Mediterranean
162	F	C	(1-10)	NA	Constantine	Suburban	Mediterranean
163	F	<B	(1-10)	NA	Constantine	Suburban	Mediterranean
164	M	<B	(1-10)	0	Constantine	Suburban	Mediterranean
165	M	<B	(11-30)	0	Constantine	Rural	Mediterranean
166	M	B	(11-30)	0	Constantine	Rural	Mediterranean
167	F	C	(11-30)	A	Constantine	Rural	Mediterranean
168	F	B	(11-30)	NA	Constantine	Rural	Mediterranean
169	F	B	(11-30)	NA	Constantine	Rural	Mediterranean
170	M	C	(11-30)	0	Constantine	Rural	Mediterranean
171	M	B	(11-30)	0	Constantine	Rural	Mediterranean
172	F	B	(11-30)	NA	Constantine	Rural	Mediterranean
173	F	<B	(11-30)	NA	Constantine	Rural	Mediterranean
174	F	C	(11-30)	NA	Constantine	Rural	Mediterranean
175	M	C	(11-30)	0	Constantine	Rural	Mediterranean
176	F	C	(11-30)	NA	Constantine	Rural	Mediterranean
177	F	C	(11-30)	NA	Constantine	Rural	Mediterranean
178	M	<B	(11-30)	0	Constantine	Rural	Mediterranean
179	F	C	(11-30)	NA	Constantine	Rural	Mediterranean
180	F	C	(1-10)	NA	Constantine	Rural	Mediterranean
181	F	B	(1-10)	NA	Constantine	Rural	Mediterranean
182	M	C	(1-10)	NA	Constantine	Rural	Mediterranean
183	M	<B	(1-10)	NA	Constantine	Rural	Mediterranean
184	F	B	(1-10)	NA	Constantine	Rural	Mediterranean
185	F	<B	(11-30)	NA	Constantine	Rural	Mediterranean
186	F	C	(11-30)	NA	Constantine	Rural	Mediterranean
187	F	B	(11-30)	A	Constantine	Rural	Mediterranean
188	M	C	(11-30)	0	Constantine	Rural	Mediterranean
189	F	<B	(11-30)	NA	Constantine	Rural	Mediterranean
190	M	C	(11-30)	0	Constantine	Rural	Mediterranean
191	F	<B	(1-10)	NA	Mila	Suburban	Mediterranean
192	F	B	(1-10)	A	Mila	Suburban	Mediterranean
193	F	B	(1-10)	A	Mila	Suburban	Mediterranean
194	F	B	(1-10)	A	Mila	Suburban	Mediterranean
195	M	<B	(1-10)	0	Mila	Suburban	Mediterranean
196	M	C	(11-30)	0	Constantine	Rural	Mediterranean
197	F	B	(11-30)	NA	Constantine	Rural	Mediterranean
198	F	B	(11-30)	A	Constantine	Rural	Mediterranean
199	M	<B	(11-30)	0	Constantine	Rural	Mediterranean

200	F	<B	(11-30)	A	Constantine	Suburban	Mediterranean
201	M	B	(11-30)	0	Constantine	Suburban	Mediterranean
202	F	B	(11-30)	NA	Constantine	Suburban	Mediterranean
203	M	C	(11-30)	0	Constantine	Suburban	Mediterranean
204	M	B	(11-30)	0	Constantine	Suburban	Mediterranean
205	F	<B	(11-30)	NA	Constantine	Suburban	Mediterranean
206	M	<B	(11-30)	0	Constantine	Suburban	Mediterranean
207	F	<B	(11-30)	0	Constantine	Suburban	Mediterranean
208	F	B	(11-30)	A	Constantine	Suburban	Mediterranean
209	F	B	(11-30)	NA	Constantine	Rural	Mediterranean
210	F	<B	(11-30)	NA	Constantine	Rural	Mediterranean
211	F	<B	(11-30)	NA	Constantine	Rural	Mediterranean
212	M	B	(11-30)	0	Constantine	Rural	Mediterranean
213	M	B	(11-30)	0	Constantine	Rural	Mediterranean
214	F	<B	(11-30)	A	Constantine	Rural	Mediterranean
215	F	<B	(11-30)	NA	Constantine	Rural	Mediterranean
216	F	<B	(1-10)	NA	Constantine	Rural	Mediterranean
217	F	B	(1-10)	NA	Constantine	Rural	Mediterranean
218	M	B	(1-10)	0	Constantine	Rural	Mediterranean
219	M	<B	(1-10)	0	Constantine	Rural	Mediterranean
220	F	B	(1-10)	A	Constantine	Rural	Mediterranean
221	M	<B	(1-10)	0	Constantine	Rural	Mediterranean
222	F	<B	(1-10)	NA	Mila	Rural	Mediterranean
223	F	B	(1-10)	NA	Mila	Rural	Mediterranean
224	F	B	(1-10)	A	Mila	Rural	Mediterranean
225	M	C	(1-10)	0	Mila	Rural	Mediterranean
226	F	B	(1-10)	NA	Mila	Rural	Mediterranean
227	M	B	(1-10)	0	Mila	Rural	Mediterranean
228	F	B	(1-10)	A	Mila	Rural	Mediterranean
229	F	B	(11-30)	NA	Mila	Rural	Mediterranean
230	F	<B	(11-30)	NA	Mila	Rural	Mediterranean
231	F	<B	(11-30)	NA	Mila	Rural	Mediterranean
232	M	<B	(11-30)	0	Mila	Rural	Mediterranean
233	F	B	(11-30)	NA	Mila	Rural	Mediterranean
234	F	B	(11-30)	A	Mila	Rural	Mediterranean
235	F	<B	(11-30)	A	Constantine	Suburban	Mediterranean
236	F	<B	(11-30)	NA	Constantine	Suburban	Mediterranean
237	M	<B	(11-30)	0	Constantine	Suburban	Mediterranean
238	F	B	(11-30)	NA	Constantine	Suburban	Mediterranean
239	F	B	(11-30)	NA	Constantine	Suburban	Mediterranean
240	M	B	(11-30)	0	Constantine	Suburban	Mediterranean
241	F	<B	(1-10)	NA	Constantine	Rural	Mediterranean
242	M	B	(1-10)	0	Constantine	Rural	Mediterranean
243	M	B	(1-10)	0	Constantine	Rural	Mediterranean
244	F	B	(1-10)	NA	Constantine	Rural	Mediterranean
245	M	<B	(11-30)	0	Constantine	Rural	Mediterranean
246	F	<B	(11-30)	A	Constantine	Rural	Mediterranean
247	F	B	(11-30)	NA	Constantine	Rural	Mediterranean
248	F	B	(11-30)	A	Constantine	Rural	Mediterranean
249	F	B	(11-30)	NA	Constantine	Rural	Mediterranean

250	F	B	(11-30)	NA	Mila	Rural	Mediterranean
251	F	<B	(11-30)	A	Mila	Rural	Mediterranean
252	F	<B	(11-30)	A	Mila	Rural	Mediterranean
253	M	B	(11-30)	0	Mila	Rural	Mediterranean
254	M	B	(11-30)	0	Mila	Rural	Mediterranean
255	F	B	(11-30)	NA	Mila	Rural	Mediterranean
256	F	<B	(11-30)	NA	Mila	Rural	Mediterranean
257	F	<B	(1-10)	NA	Mila	Suburban	Mediterranean
258	M	B	(1-10)	0	Mila	Suburban	Mediterranean
259	M	C	(1-10)	0	Mila	Suburban	Mediterranean
260	F	<B	(1-10)	A	Mila	Suburban	Mediterranean
261	F	C	(1-10)	NA	Mila	Suburban	Mediterranean
262	M	C	(1-10)	0	Mila	Suburban	Mediterranean
263	F	B	(1-10)	NA	Mila	Suburban	Mediterranean
264	F	B	(11-30)	NA	Mila	Rural	Mediterranean
265	F	<B	(11-30)	NA	Mila	Rural	Mediterranean
266	M	<B	(11-30)	0	Mila	Rural	Mediterranean
267	F	B	(11-30)	A	Mila	Rural	Mediterranean
268	F	B	(11-30)	NA	Mila	Rural	Mediterranean
269	F	B	(11-30)	NA	Mila	Rural	Mediterranean
270	M	<B	(11-30)	0	Mila	Rural	Mediterranean
271	M	B	(11-30)	0	Mila	Rural	Mediterranean
272	F	C	(11-30)	A	Mila	Rural	Mediterranean
273	M	<B	(1-10)	0	Mila	Rural	Mediterranean
274	F	B	(1-10)	NA	Mila	Rural	Mediterranean
275	M	<B	(1-10)	0	Mila	Rural	Mediterranean
276	F	B	(1-10)	NA	Mila	Rural	Mediterranean
277	F	B	(1-10)	A	Mila	Rural	Mediterranean
278	F	B	(1-10)	NA	Mila	Rural	Mediterranean
279	M	<B	(1-10)	0	Mila	Rural	Mediterranean
280	M	B	(1-10)	0	Constantine	Rural	Mediterranean
281	F	B	(1-10)	NA	Constantine	Rural	Mediterranean
282	F	<B	(1-10)	NA	Constantine	Rural	Mediterranean
283	M	<B	(1-10)	0	Constantine	Rural	Mediterranean
284	F	B	(1-10)	A	Constantine	Rural	Mediterranean
285	F	C	(1-10)	NA	Constantine	Rural	Mediterranean
286	F	C	(11-30)	A	Constantine	Rural	Mediterranean
287	F	B	(11-30)	NA	Constantine	Rural	Mediterranean
288	M	<B	(11-30)	0	Constantine	Rural	Mediterranean
289	M	<B	(11-30)	0	Constantine	Rural	Mediterranean
290	F	<B	(11-30)	NA	Constantine	Rural	Mediterranean
291	F	B	(11-30)	NA	Constantine	Rural	Mediterranean
292	F	B	(11-30)	A	Constantine	Rural	Mediterranean
293	M	B	(11-30)	0	Constantine	Rural	Mediterranean
294	F	C	(11-30)	NA	Constantine	Rural	Mediterranean
295	F	B	(11-30)	NA	Mila	Rural	Mediterranean
296	M	B	(11-30)	0	Mila	Rural	Mediterranean
297	F	<B	(11-30)	NA	Mila	Rural	Mediterranean
298	F	<B	(11-30)	A	Mila	Rural	Mediterranean
299	M	<B	(11-30)	0	Mila	Rural	Mediterranean

300	M	<B	(11-30)	0	Mila	Rural	Mediterranean
301	F	B	(1-10)	A	Mila	Rural	Mediterranean
302	F	B	(1-10)	A	Mila	Rural	Mediterranean
303	M	<B	(1-10)	0	Mila	Rural	Mediterranean
304	F	<B	(1-10)	NA	Mila	Rural	Mediterranean
305	F	B	(1-10)	A	Mila	Rural	Mediterranean
306	F	B	(1-10)	NA	Mila	Rural	Mediterranean
307	F	<B	(1-10)	A	Constantine	Rural	Mediterranean
308	F	B	(1-10)	NA	Constantine	Rural	Mediterranean
309	M	<B	(1-10)	0	Constantine	Rural	Mediterranean
310	F	B	(1-10)	A	Constantine	Rural	Mediterranean
311	F	B	(1-10)	NA	Constantine	Rural	Mediterranean
312	M	<B	(1-10)	0	Constantine	Rural	Mediterranean
313	F	B	(1-10)	A	Constantine	Rural	Mediterranean
314	F	<B	(1-10)	A	Constantine	Rural	Mediterranean
315	F	B	(1-10)	A	Constantine	Rural	Mediterranean
316	F	B	(1-10)	NA	Constantine	Rural	Mediterranean
317	F	B	(1-10)	NA	Constantine	Rural	Mediterranean
318	M	<B	(1-10)	0	Constantine	Rural	Mediterranean
319	F	B	(1-10)	NA	Mila	Rural	Mediterranean
320	F	B	(1-10)	A	Mila	Rural	Mediterranean
321	M	B	(1-10)	0	Mila	Rural	Mediterranean
322	F	C	(1-10)	NA	Constantine	Rural	Mediterranean
323	F	B	(1-10)	A	Constantine	Rural	Mediterranean
324	M	B	(1-10)	0	Constantine	Rural	Mediterranean
325	F	<B	(1-10)	A	Constantine	Rural	Mediterranean
326	F	B	(1-10)	NA	Constantine	Rural	Mediterranean
327	F	C	(1-10)	NA	Constantine	Rural	Mediterranean
328	F	B	(11-30)	A	Constantine	Rural	Mediterranean
329	F	<B	(11-30)	NA	Constantine	Rural	Mediterranean
330	F	B	(11-30)	NA	Constantine	Rural	Mediterranean
331	F	B	(11-30)	A	Constantine	Rural	Mediterranean
332	F	C	(11-30)	NA	Constantine	Rural	Mediterranean
333	F	B	(11-30)	NA	Constantine	Rural	Mediterranean
334	M	B	(11-30)	0	Constantine	Rural	Mediterranean
335	F	<B	(11-30)	NA	Constantine	Suburban	Mediterranean
336	F	B	(11-30)	NA	Constantine	Suburban	Mediterranean
337	M	B	(11-30)	0	Constantine	Suburban	Mediterranean
338	F	B	(11-30)	A	Constantine	Suburban	Mediterranean
339	F	<B	(11-30)	NA	Constantine	Suburban	Mediterranean
340	M	<B	(11-30)	0	Constantine	Suburban	Mediterranean
341	M	<B	(11-30)	0	Constantine	Suburban	Mediterranean
342	F	B	(1-10)	A	Mila	Rural	Mediterranean
343	F	<B	(1-10)	A	Mila	Rural	Mediterranean
344	F	B	(1-10)	NA	Mila	Rural	Mediterranean
345	F	B	(1-10)	A	Mila	Rural	Mediterranean
346	F	B	(1-10)	NA	Mila	Rural	Mediterranean
347	F	<B	(1-10)	NA	Mila	Rural	Mediterranean
348	F	B	(1-10)	A	Mila	Rural	Mediterranean
349	M	C	(1-10)	0	Mila	Rural	Mediterranean

350	F	C	(1-10)	NA	Constantine	Rural	Mediterranean
351	F	<B	(1-10)	NA	Constantine	Rural	Mediterranean
352	F	B	(1-10)	A	Constantine	Rural	Mediterranean
353	M	<B	(1-10)	0	Constantine	Rural	Mediterranean
354	M	B	(1-10)	0	Constantine	Rural	Mediterranean
355	F	C	(11-30)	A	Mila	Rural	Mediterranean
356	F	B	(11-30)	NA	Mila	Rural	Mediterranean
357	F	B	(11-30)	NA	Mila	Rural	Mediterranean
358	F	<B	(11-30)	A	Mila	Rural	Mediterranean
359	F	<B	(11-30)	NA	Mila	Rural	Mediterranean
360	M	B	(11-30)	0	Mila	Rural	Mediterranean
361	F	<B	(11-30)	NA	Guelma	Suburban	Sub-humid
362	F	<B	(11-30)	NA	Guelma	Suburban	Sub-humid
363	F	C	(11-30)	NA	Guelma	Suburban	Sub-humid
364	M	B	(11-30)	0	Guelma	Suburban	Sub-humid
365	M	B	(11-30)	0	Guelma	Suburban	Sub-humid
366	F	<B	(11-30)	NA	Guelma	Suburban	Sub-humid
367	F	B	(+31)	A	El-Taref	Suburban	Humid
368	F	<B	(+31)	NA	El-Taref	Suburban	Humid
369	M	<B	(+31)	0	El-Taref	Suburban	Humid
370	M	C	(+31)	0	El-Taref	Suburban	Humid
371	F	<B	(+31)	A	El-Taref	Suburban	Humid
372	M	B	(+31)	0	El-Taref	Suburban	Humid
373	F	<B	(+31)	NA	El-Taref	Suburban	Humid
374	F	C	(11-30)	NA	Guelma	Rural	Sub-humid
375	M	<B	(11-30)	0	Guelma	Rural	Sub-humid
376	F	B	(11-30)	NA	Guelma	Rural	Sub-humid
377	M	B	(11-30)	0	Guelma	Rural	Sub-humid
378	F	<B	(11-30)	A	Guelma	Rural	Sub-humid
379	M	<B	(11-30)	0	Guelma	Rural	Sub-humid
380	F	<B	(11-30)	NA	Guelma	Rural	Sub-humid
381	F	B	(11-30)	A	Guelma	Rural	Sub-humid
382	M	<B	(11-30)	0	El-Taref	Rural	Humid
383	M	<B	(11-30)	0	El-Taref	Rural	Humid
384	M	<B	(11-30)	0	El-Taref	Rural	Humid
385	M	<B	(11-30)	0	El-Taref	Rural	Humid
386	M	<B	(11-30)	0	El-Taref	Rural	Humid
387	M	<B	(11-30)	0	El-Taref	Rural	Humid
388	M	<B	(11-30)	0	El-Taref	Rural	Humid
389	M	<B	(11-30)	0	El-Taref	Rural	Humid
390	M	C	(11-30)	0	El-Taref	Rural	Humid
391	M	<B	(11-30)	0	El-Taref	Rural	Humid
392	F	C	(11-30)	A	Guelma	Rural	Sub-humid
393	F	B	(11-30)	NA	Guelma	Rural	Sub-humid
394	F	B	(1-10)	NA	Guelma	Rural	Sub-humid
395	M	C	(1-10)	0	Guelma	Rural	Sub-humid
396	F	B	(1-10)	NA	Guelma	Rural	Sub-humid
397	F	<B	(1-10)	NA	Guelma	Rural	Sub-humid
398	M	C	(11-30)	0	Guelma	Rural	Sub-humid
399	F	C	(11-30)	NA	Guelma	Rural	Sub-humid

400	M	<B	(11-30)	0	Guelma	Rural	Sub-humid
401	F	<B	(11-30)	NA	Guelma	Rural	Sub-humid
402	F	B	(11-30)	NA	Guelma	Rural	Sub-humid
403	F	B	(11-30)	NA	Guelma	Rural	Sub-humid
404	F	<B	(11-30)	NA	Guelma	Rural	Sub-humid
405	M	<B	(11-30)	0	Guelma	Rural	Sub-humid
406	M	C	(11-30)	0	Guelma	Rural	Sub-humid
407	F	<B	(11-30)	A	Guelma	Rural	Sub-humid
408	F	B	(11-30)	A	Guelma	Rural	Sub-humid
409	M	B	(11-30)	0	Guelma	Rural	Sub-humid
410	F	B	(11-30)	A	Guelma	Rural	Sub-humid
411	F	C	(11-30)	NA	Guelma	Rural	Sub-humid
412	F	B	(11-30)	A	Guelma	Rural	Sub-humid
413	F	B	(11-30)	NA	Guelma	Rural	Sub-humid
414	F	B	(11-30)	NA	Guelma	Rural	Sub-humid
415	F	<B	(11-30)	A	Guelma	Rural	Sub-humid
416	F	B	(11-30)	A	Guelma	Rural	Sub-humid
417	F	<B	(11-30)	NA	Guelma	Rural	Sub-humid
418	F	<B	(11-30)	NA	Guelma	Rural	Sub-humid
419	M	<B	(11-30)	0	Guelma	Rural	Sub-humid
420	M	<B	(11-30)	0	Guelma	Rural	Sub-humid
421	M	<B	(11-30)	0	Guelma	Rural	Sub-humid
422	M	B	(11-30)	0	El-Taref	Rural	Humid
423	M	B	(11-30)	0	El-Taref	Rural	Humid
424	F	<B	(11-30)	NA	El-Taref	Rural	Humid
425	F	B	(11-30)	NA	El-Taref	Rural	Humid
426	M	<B	(11-30)	0	El-Taref	Rural	Humid
427	M	<B	(11-30)	0	El-Taref	Rural	Humid
428	F	C	(11-30)	NA	El-Taref	Rural	Humid
429	F	<B	(11-30)	NA	Guelma	Rural	Sub-humid
430	F	B	(11-30)	NA	Guelma	Rural	Sub-humid
431	F	<B	(11-30)	NA	Guelma	Rural	Sub-humid
432	F	C	(11-30)	NA	Guelma	Rural	Sub-humid
433	F	B	(11-30)	A	Guelma	Rural	Sub-humid
434	F	<B	(11-30)	NA	Guelma	Rural	Sub-humid
435	F	B	(11-30)	A	Guelma	Rural	Sub-humid
436	F	C	(11-30)	A	Guelma	Rural	Sub-humid
437	M	B	(11-30)	0	Guelma	Rural	Sub-humid
438	F	<B	(1-10)	NA	Guelma	Rural	Sub-humid
439	M	<B	(1-10)	0	Guelma	Rural	Sub-humid
440	M	B	(1-10)	0	Guelma	Rural	Sub-humid
441	F	B	(1-10)	A	Guelma	Rural	Sub-humid
442	F	B	(1-10)	A	Guelma	Rural	Sub-humid
443	F	B	(1-10)	A	Guelma	Rural	Sub-humid
444	F	B	(1-10)	A	Guelma	Rural	Sub-humid
445	F	C	(1-10)	A	Guelma	Rural	Sub-humid
446	F	B	(1-10)	NA	Guelma	Rural	Sub-humid
447	M	<B	(1-10)	0	Guelma	Rural	Sub-humid
448	F	B	(1-10)	NA	Guelma	Rural	Sub-humid
449	F	B	(1-10)	NA	Guelma	Rural	Sub-humid

450	M	<B	(11-30)	0	Guelma	Rural	Sub-humid
451	F	<B	(11-30)	NA	Guelma	Rural	Sub-humid
452	F	C	(11-30)	NA	Guelma	Rural	Sub-humid
453	F	C	(11-30)	A	Guelma	Rural	Sub-humid
454	F	B	(11-30)	A	Guelma	Rural	Sub-humid
455	F	B	(11-30)	NA	Guelma	Rural	Sub-humid
456	M	C	(11-30)	0	Guelma	Rural	Sub-humid
457	M	B	(11-30)	0	Guelma	Rural	Sub-humid
458	F	C	(11-30)	A	Guelma	Rural	Sub-humid
459	F	C	(11-30)	A	Guelma	Rural	Sub-humid
460	F	B	(11-30)	A	Guelma	Rural	Sub-humid
461	F	B	(11-30)	A	Guelma	Rural	Sub-humid
462	F	<B	(11-30)	NA	Guelma	Rural	Sub-humid
463	M	<B	(11-30)	0	Guelma	Rural	Sub-humid
464	M	<B	(11-30)	0	Guelma	Rural	Sub-humid
465	F	B	(11-30)	A	Guelma	Rural	Sub-humid
466	F	B	(11-30)	A	Guelma	Rural	Sub-humid
467	F	B	(11-30)	A	Guelma	Rural	Sub-humid
468	F	B	(11-30)	A	Guelma	Rural	Sub-humid
469	F	B	(11-30)	A	Guelma	Rural	Sub-humid
470	F	<B	(1-10)	NA	Guelma	Rural	Sub-humid
471	M	<B	(1-10)	0	Guelma	Rural	Sub-humid
472	M	B	(1-10)	0	Guelma	Rural	Sub-humid
473	F	<B	(1-10)	NA	Guelma	Rural	Sub-humid
474	F	<B	(1-10)	NA	Guelma	Rural	Sub-humid
475	F	<B	(1-10)	NA	Guelma	Rural	Sub-humid
476	F	<B	(1-10)	NA	Guelma	Rural	Sub-humid
477	F	B	(1-10)	NA	Guelma	Rural	Sub-humid
478	F	B	(11-30)	NA	Guelma	Rural	Sub-humid
479	F	B	(11-30)	NA	Guelma	Rural	Sub-humid
480	M	B	(11-30)	0	Guelma	Rural	Sub-humid
481	M	<B	(11-30)	0	Guelma	Rural	Sub-humid
482	M	<B	(11-30)	0	Guelma	Rural	Sub-humid
483	F	B	(11-30)	NA	Guelma	Rural	Sub-humid
484	F	B	(11-30)	NA	Guelma	Rural	Sub-humid
485	F	B	(11-30)	NA	Guelma	Rural	Sub-humid
486	M	<B	(1-10)	0	Guelma	Rural	Sub-humid
487	M	<B	(1-10)	0	Guelma	Rural	Sub-humid
488	M	<B	(1-10)	0	Guelma	Rural	Sub-humid
489	F	<B	(1-10)	NA	Guelma	Rural	Sub-humid
490	M	<B	(1-10)	0	Guelma	Rural	Sub-humid
491	F	<B	(1-10)	NA	Guelma	Rural	Sub-humid
492	F	B	(1-10)	NA	Guelma	Rural	Sub-humid
493	F	B	(1-10)	NA	Guelma	Rural	Sub-humid
494	F	<B	(1-10)	A	Guelma	Rural	Sub-humid
495	F	B	(1-10)	A	Guelma	Rural	Sub-humid
496	F	<B	(1-10)	NA	Guelma	Rural	Sub-humid
497	M	B	(1-10)	0	Guelma	Rural	Sub-humid
498	M	<B	(1-10)	0	Guelma	Rural	Sub-humid
499	F	<B	(1-10)	0	Guelma	Rural	Sub-humid

500	M	C	(1-10)	0	Guelma	Rural	Sub-humid
501	F	<B	(1-10)	NA	Guelma	Rural	Sub-humid
502	F	B	(1-10)	NA	Guelma	Rural	Sub-humid
503	F	<B	(11-30)	A	El-Taref	Rural	Humid
504	M	<B	(11-30)	0	El-Taref	Rural	Humid

M – Male; F – Female; A – Abortion; NA - No Abortion; <B - animals under 2 years of age; B - animals between 2 and 5 years ; C - animals over 5 years of age
